# An Exploration on Trust

**DOI:** 10.1093/geroni/igab046.019

**Published:** 2021-12-17

**Authors:** Elena Portacolone

## Abstract

“Trust is a form of love,” explained a study participant. As a form of love, trust nourishes connections and accelerates progress. As a result, the purpose of this session is to reflect upon the notion of trust and examine how trust moves science and social justice forward. Trust must be seen as sustained or broken over multiple generations. Moreover, trust between older adults and medical and social support institutions has profound implications for this historical moment. In the COVID-19 pandemic, trust can be viewed as a facilitator of emergency responses in the State of Washington as noted in Dr. Berridge’s paper. On the other hand, distrust and a related sense of abandonment contributes to Black Americans' limited uptake of COVID-19 vaccinations, as noted in Dr. Johnson’s work. On a related note, Dr. Perry’s work shows that lack of trust over time has led those aging with hemophilia to withdraw from care at different points in their own trajectories. Finally, on a positive note, Dr. Kotwal’s work illustrates the role of a peer outreach intervention in facilitating trusting relationships among diverse, low-income older adults which led to sustained reductions, over a 2-year period, in loneliness, barriers to socializing, and depression. This symposium on trust highlights how researchers work, either consciously or unconsciously, within a continuum of trust in their participants' communities. At a broader level, systemic attention to building trust from academia, government, and national advocacy organizations holds the potential to foster meaningful scientific engagement and empowerment of historically marginalized communities.

